# Supplementary material for: Familial Risk and Heritability of Hematologic Malignancies in the Nordic Twin Study of Cancer
Source: Cancers (Basel). 2021 Jun 16;13(12):3023. doi: 10.3390/cancers13123023 (PMC8234145; doi:10.3390/cancers13123023)
Supplement: Supplementary file 1 [file cancers-13-03023-s001.zip › cancers-1238933-supplementary.pdf]

cancers MDPI

Supplementary Materials: Familial Risk and Heritability of Hematologic Malignancies in the Nordic Twin Study of Cancer

Signe B. Clemmensen, Jennifer R. Harris, Jonas Mengel-From, Wagner H. Bonat, Henrik Frederiksen, Jaakko Kaprio and Jacob v. B. Hjelmborg

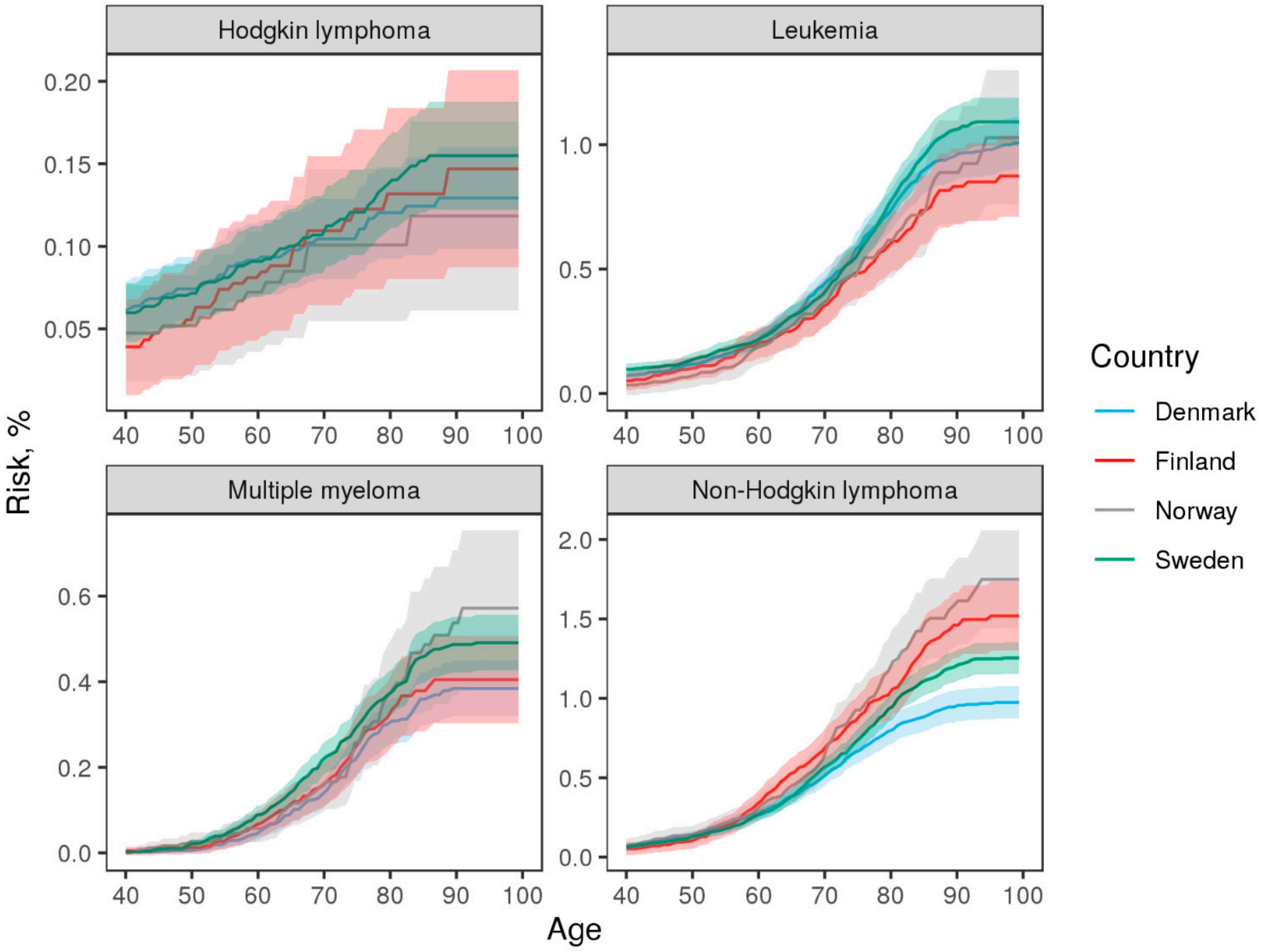

Figure S1. Cumulative incidence and 95% confidence intervals for hematologic malignancies by age in each of the Nordic countries, adjusted for censoring and competing risk of death.

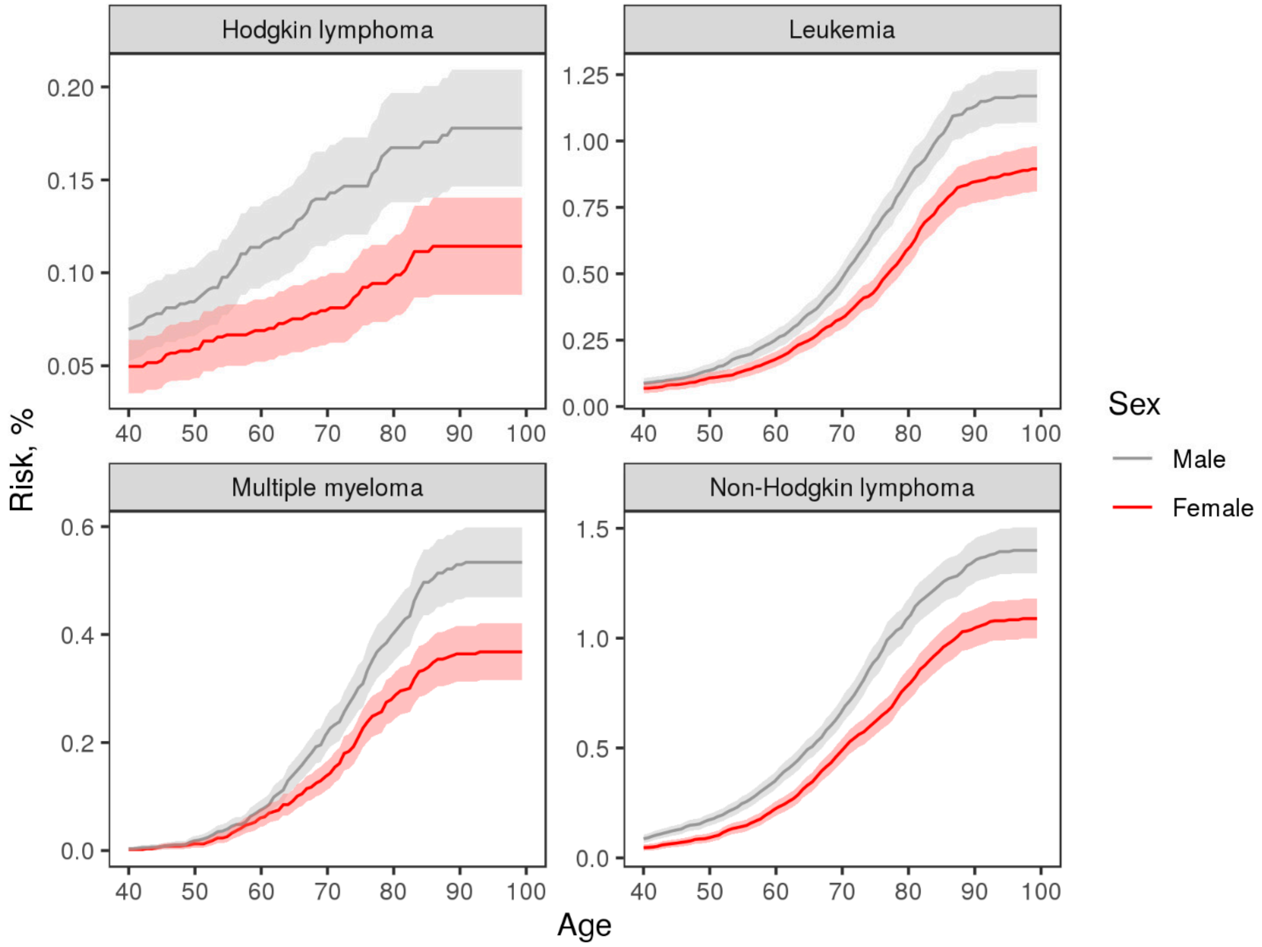

Figure S2. Cumulative incidence and 95% confidence intervals for hematologic malignancies by age and sex, adjusted for censoring and competing risk of death.

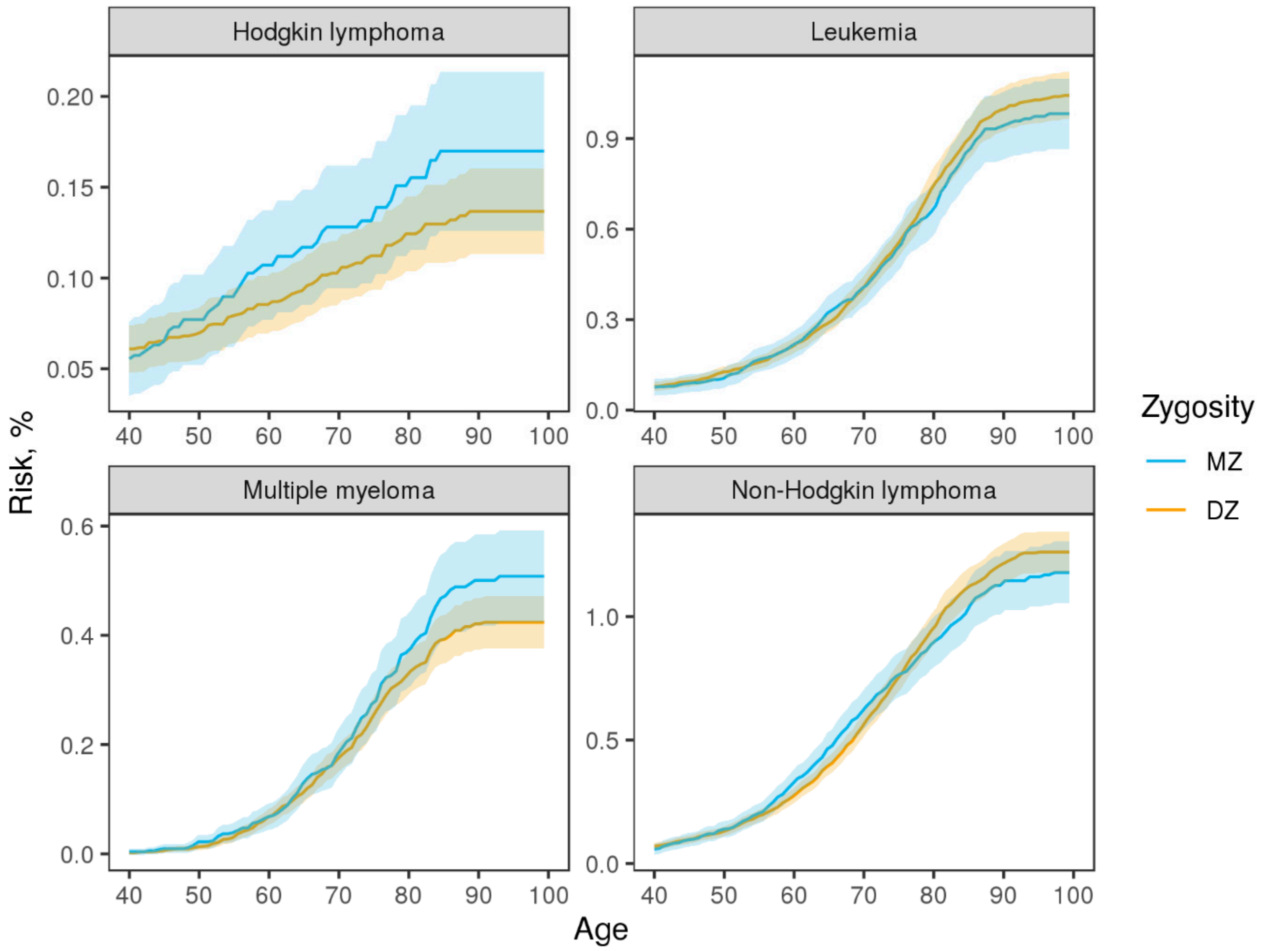

Figure S3. Cumulative incidence and 95% confidence intervals for hematologic malignancies by age and zygosity, adjusted for censoring and competing risk of death.

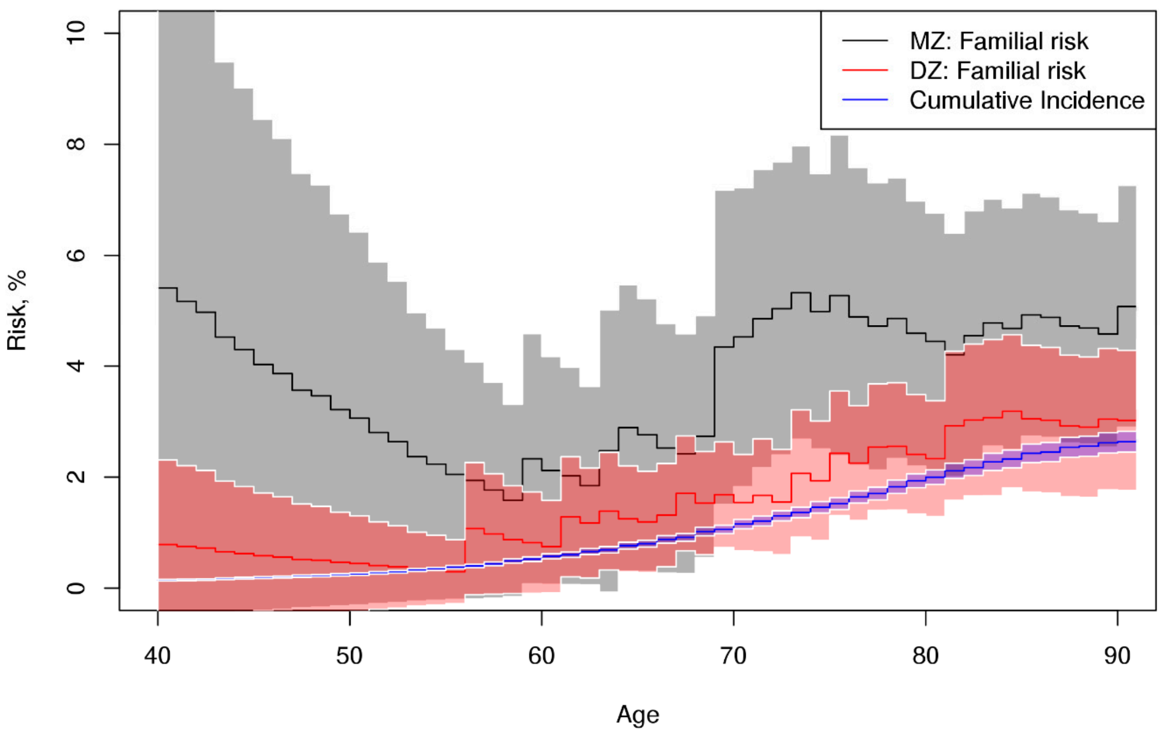

Figure S4. Cumulative incidence and familial risk for monozygotic (MZ) and dizygotic (DZ) twins by age and 95% confidence intervals for overall hematologic malignancy in the NorTwinCan cohort, adjusted for censoring and competing risk of death.

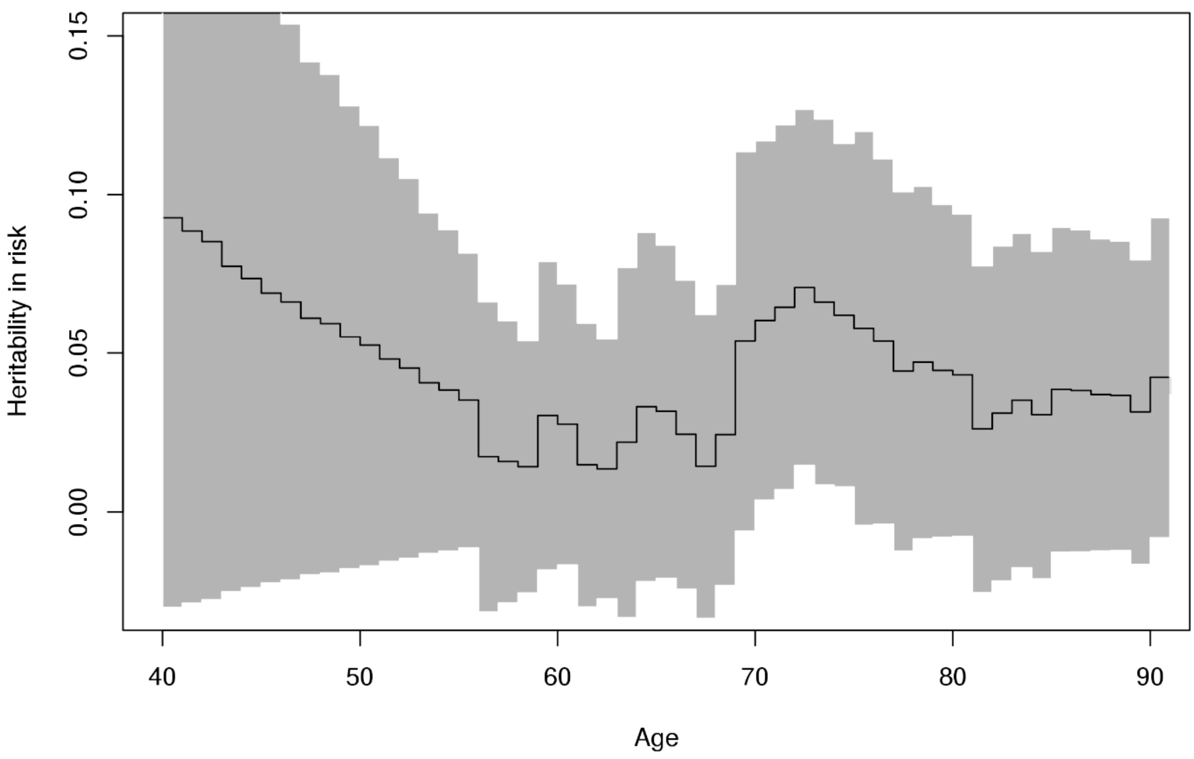

Figure S5. Heritability in risk by age and 95% confidence interval for overall hematologic malignancy in the NorTwinCan cohort, adjusted for censoring and competing risk of death.

Table S1. Number of pairs where one twin was diagnosed with a hematologic malignancy and the co-twin was diagnosed with another type of cancer (following the NORDCAN classification). Estimates of lifetime relative recurrence risk and heritability at age 100 among twins in the NorTwinCan cohort, adjusted for censoring and competing risk of death.

Hematologic Malignancy	Number of Twin Pairs		Relative Recurrence Risk (95% CI)		Heritability in Risk, % (95% CI)
	MZ	DZ	MZ	DZ	
Lip, oral cavity and pharynx	9	14	3.0 (1.0-5.1)	1.7 (1.2-2.1)	4.0 (-2.8-10.9)
Esophagus	4	8	2.2 (0.0-4.5)	2.1 (0.6-3.6)	0.4 (-5.9-6.8)
Stomach	6	12	1.6 (0.2-3.1)	1.0 (0.4-1.7)	2.2 (-3.8-8.3)
Small intestine	0	4	-	-	-
Colon	20	46	2.1 (1.1-3.1)	1.6 (1.1-2.2)	2.7 (-3.9-9.4)
Rectum and anus	9	25	1.2 (0.3-2.0)	1.4 (0.8-2.0)	-1.2 (-6.1-3.7)
Liver	4	9	2.2 (0.0-4.5)	1.9 (0.6-3.2)	0.8 (-5.5-7.1)
Gallbladder	5	7	3.3 (0.3-6.2)	1.7 (0.4-3.0)	3.7 (-3.6-11.0)
Pancreas	8	12	1.9 (0.5-3.3)	1.4 (0.6-2.2)	1.9 (-3.9-7.7)
Nose, sinuses	0	0	-	-	-
Larynx	1-3	5	2.2 (-1.0-5.5)	1.7 (0.2-3.3)	0.9 (-5.3-7.1)
Lung	22	54	1.9 (1.1-2.8)	1.6 (1.2-2.1)	1.8 (-4.6-8.2)
Pleura	0	1-3	-	-	-
Bone	1-3	0	-	-	-
Melanoma of skin	9	28	1.4 (0.4-2.3)	1.8 (1.1-2.5)	-1.8 (-7.3-3.6)
Skin, non-melanoma	24	50	2.5 (1.5-3.6)	1.9 (1.3-2.4)	4.4 (-3.5-12.3)
Soft tissues	1-3	1-3	2.2 (-0.9-5.3)	1.1 (-0.7-2.8)	1.9 (-3.8-7.6)
Breast [1]	34	86	1.7 (1.1-2.4)	1.5 (1.0-1.9)	2.9 (-5.7-11.4)
Cervix uteri [1]	11	34	1.2 (0.4-2.0)	1.0 (0.4-1.6)	1.4 (-5.3-8.1)
Corpus uteri [1]	6	12	1.4 (0.2-2.6)	0.8 (0.1-1.5)	3.1 (-3.6-9.7)
Uterus, other [1]	0	1-3	-	-	-
Ovary [1]	5	15	1.6 (0.1-3.1)	1.1 (0.3-2.0)	2.0 (-5.7-9.7)
Other female genital organs [1]	0	1-3	-	-	-
Prostate [2]	45	106	2.5 (1.7-3.2)	2.3 (1.7-2.8)	2.5 (-8.9-13.9)
Testis [2]	1-3	4	5.0 (-2.3-12.3)	1.3 (-0.2-2.9)	8.8 (-9.0-26.7)
Penis [2]	0	1-3	-	-	-
Kidney	6	16	2.0 (0.4-3.7)	2.2 (1.1-3.4)	-0.7 (-6.9-5.5)
Bladder	18	36	2.3 (1.1-3.4)	2.0 (1.3-2.6)	1.6 (-5.3-8.5)
Eye	0	1-3	-	-	-
Brain, central nervous system	7	26	1.5 (0.2-2.8)	2.0 (1.2-2.8)	-1.9 (-7.8-4.1)
Thyroid	1-3	1-3	1.2 (-0.5-2.9)	1.0 (-0.2-2.3)	0.3 (-3.6-4.1)

[1] Females only.
[2] Males only.
